# Tissue Resident Memory γδT Cells in Murine Uterus Expressed High Levels of IL-17 Promoting the Invasion of Trophocytes

**DOI:** 10.3389/fimmu.2020.588227

**Published:** 2021-01-14

**Authors:** Shuangpeng Kang, Qiongli Wu, Jun Huang, Binyan Yang, Changyan Liang, Peidong Chi, Changyou Wu

**Affiliations:** ^1^ Institute of Immunology, Zhongshan School of Medicine, Sun Yat-sen University, Guangzhou, China; ^2^ Key Laboratory of Immunology, Sino-French Hoffmann Institute, Guangzhou Medical University, Guangzhou, China; ^3^ Department of Gynecology and Obstetrics, Third Affiliated Hospital of Sun Yat-Sen University, Guangzhou, China; ^4^ Department of Clinical Laboratory, Sun Yat-sen University Cancer Center, State Key Laboratory of Oncology in South China, Collaborative Innovation Center for Cancer Medicine, Guangzhou, China

**Keywords:** tissue resident memory γδT cell, uterus, IL-17, RORγt, trophocyte

## Abstract

γδT cells are non-conventional T cells and serve as the bridge for connecting the innate and adaptive immune systems. γδT cells form a substantial population at barrier sites and play an important role in the development of physiology, inflammation, autoimmune diseases and tumors. γδT cells not only distribute in the maternal-fetal interface during pregnancy but also in non-pregnant uterus. However, the phenotypes and functions of γδT cells in uterus were not clear. In the current study, we found that the percentages of γδT cells were significantly higher in uterus than peripheral blood and most of γδT cells in uterus were distributed in endometrium. Further studies indicated that the majority of γδT cells in uterus were memory cells with higher expression of CD44 and CD27 but lower expression of CD62L and CCR7 compared to those in blood. In addition, we found that γδT cells in uterus were tissue resident memory γδT cells expressing CD69, expressed high levels of CCR6, GranzymeB and CD107a. Moreover, γδT cells in uterus were activated and fully expressed transcription factor RORγt. After short time of activation, γδT cells in uterus significantly expressed high levels of IL-17 but not IFN-γ, which promotes the invasion of murine trophocytes. Taken together, our study will lay the foundation for future research on uterine γδT cells in pregnancy and autoimmune disease.

## Introduction

T cells can be divided into αβT cells and γδT cells according to the constitution of T cell receptor (TCR). Unlike αβT cells, γδT cells are greatly enriched in mucosal and epithelial sites, such as the skin, respiratory tract, reproductive tracts and display immunologic features common to both the innate and adaptive immune systems (1–4). γδT cells can be divided into either IFN-γ-producing γδT cells or IL-17–producing γδT cells (5, 6). It has been previously reported that γδT cells play divergent roles in different tissues, such as pathogen clearance, core body temperature control in adipose and synaptic plasticity regulation in meninges (7–9). In contrast to their beneficial role, γδT cells can lead to negative outcomes or exacerbate disease in some autoimmune diseases and cancers (10–12).

It was previously thought that memory T cells consisted of two major subsets: central memory T (T_CM_) cells and effector memory T (T_EM_) cells (13). More recently, it has been clear that there is another important subset of memory T cells: tissue resident memory T (T_RM_) cells. Once established, T_RM_ cells could be long-term residency in peripheral tissues and provide protective immunity against pathogens that enter through the local tissues (14–16). In recent years, long-lived memory γδT cells are observed in certain tissues after local inflammation or bacterial infection which is termed as tissue resident γδT cells (3, 17, 18). Different from γδT cells in other tissues, the immune cells of uterus must both protect against infection and tolerate an allogeneic fetus during pregnancy. γδT cells are found to be abundant not only in the maternal-fetal interface during pregnancy, but also in non-pregnant uterus. In addition, there was higher percentages of γδT cells in allogenic matings compared with syngeneic matings, indicating that γδT cells may play an important role in successful pregnancy (4, 19, 20). γδT cells are significantly abundant in uterus and highly expressed IL-17. As we all know, autoimmune diseases tend to occur in women and IL-17 plays a crucial role in the development and progression of autoimmune diseases (11, 21, 22). Thus, it is tempting to assume that IL-17-producing-γδT cells in uterus may be related to the occurrence of autoimmune diseases in women. However, few literature on the phenotypic and functional characteristics of γδT cells in uterus has been published.

In the present study, to our knowledge for the first time, we found that γδT cells were enriched in uterus and the majority of γδT cells in uterus were tissue resident memory cells expressing significant high percentages of IL-17 with transcription factor RORγt but not IFN-γ. Based on our data, we might propose that those γδT cells in uterus were key source of IL-17, which can promote the invasion of trophocytes and play an important role in protecting infections or may be involved in the pregnancy or the pathogenesis of some autoimmune diseases in young female humans.

## Materials and Methods

### Study Participants

Human endometrium tissues from 20 individuals obtained from patients undergoing uterectomy at Third Affiliated Hospital of Sun Yat-Sen University. Written informed consent was obtained from all individuals, and the protocol was approved by the Review Board of Sun Yat-sen University.

### Animals

C57BL/6 mice aged 6 to 8 weeks were purchased from the Laboratory Animal Center of Sun Yat-sen University (S.C. XK 2016‐0029) and maintained under pathogen-free conditions. Mice were age-and weight-matched in each experiment. All animal studies were reviewed and approved by the Zhongshan School of Experimental Animal Ethics Committee, Sun Yat-sen University, Guangzhou, China.

### *In Vivo* Labeling of Cells in Circulation

To distinguish cells in circulation from uterine immune cells, anti‐CD45‐ phycoerythrin (clone: 30‐F11, BD Bioscience) was diluted to 10 μg/ml in sterile PBS and injected intravenously into mice in a volume of 200 μl/mouse. The mice were sacrificed after 5 min with or without cardiac perfusion, and mononuclear cells were isolated from blood and tissues for experiments. The cells labeled with anti‐CD45 were identified as in circulation, and its counterpart was identified as uterine immune cells in flow cytometry data.

### Cell Isolation

Mice were anesthetized by isoflurane and sacrificed in sterile environment. The blood in tissues was removed by cardiac perfusion with 50 ml PBS. Uterus were collected after opening the abdominal cavity and removing the surrounding adipose tissues. Uterus and human endometrium were cut into small pieces and digested with 5 to 10 ml digestion medium containing 2 mg/ml endotoxin-free collagenase I and 10 mg/ml DNase I (Sigma-Aldrich, USA) in RPMI-1640 for 1 h at 37°C with regular gentle shaking. Single cell suspensions were obtained by filtering through a 100-μm nylon cell strainer (BD Bioscience Pharmingen, USA). The cell suspension was carefully layered over a discontinuous (40%/70%) Percoll (GE healthcare, USA) density gradients and then centrifuged at speed 2500 rpm/min for 20 min. The middle layer mononuclear cells were collected and washed with complete RPMI-1640 twice. The cells were counted and adjusted to a density of 1×10^6^ cells/ml in complete RPMI-1640 medium for the next study.

Blood was obtained through orbit and heparin was used as anticoagulation. Blood was loaded onto Ficoll-Hypaque (Tianjin HaoYang Biological Manufacture, China) and centrifuged at speed 2200 rpm/min for 20 min, collected the mononuclear cells on upper layer and washed with complete RPMI-1640 twice. The cells were counted and adjusted to a density of 1 × 10^6^ cells/ml in complete RPMI-1640 medium for the next study.

Murine placenta were collected on 13.5 dpc in pregnancy, minced into small pieces and digested with digestion medium containing 0.5 mg/ml endotoxin-free collagenase I (Sigma- Aldrich, USA) in RPMI-1640 for 30 min at 37°C. The cell suspension was carefully layered over a discontinuous (30%/45%/60%) Percoll density gradients and then centrifuged at speed 2200 rpm/min for 20 min. Single cell suspensions were obtained between 30% and 45% Percoll gradient and cultured in 5% CO2 at 37°C for 30 min to remove the macrophage and fibroblasts. The non-adherent trophocytes were counted and adjusted to a density of 2.5 × 10^5^ cells/ml in RPMI-1640 medium.

### Flow Cytometry and MAbs

The procedures for studying cell phenotype, intracellular cytokines, and transcriptional factor expression had been previously described (23). Generally, cells were washed and suspended in 100 μl of PBS containing 0.1% BSA and 0.05% sodium azide. For surface staining, cells were incubated with the respective mAbs at 4°C in the dark for 30 min. For the detection of intracellular cytokines, cells were fixed with 4% paraformaldehyde and permeabilized in PBS buffer containing 0.1% saponin (Sigma-Aldrich, USA), 0.1% BSA and 0.05% sodium azide for at least 2 h or overnight at 4°C and stained with conjugated mAbs for intracellular cytokines. For the detection of intracellular transcription factors, cells were stained for surface antigens, followed by fixation, permeabilization with Permeabilization/Fixation buffer (BD Bioscience, USA) and staining according to the protocol of Permeabilization/Fixation Kit. Flow cytometry data were acquired with FACS Arial II (BD Bioscience, USA) and were analyzed with FlowJo software (Tree Star, USA). The following mAbs were used for cell surface or intracellular staining: PE-CF594 labeled anti-CD3, FITC labeled anti- γδT, anti-CD107a, anti-CD4, anti-CD127, APC-labeled anti-CD62L, anti-IFN-γ, anti-TNF-α, anti-NKG2D, anti-CD25, PE-labeled anti- γδT, anti- IL-17, anti-CCR7, anti-CD103, anti-GranzymeB, anti-RORγt, anti-pSTAT3, anti-Foxp3, Percp-cy5.5-labeled anti- CD44, PE-cy7 labeled anti-CD69, anti-IFN-γ, Apc-cy7 labeled anti-IL-17 and isotype-matched control antibodies were all purchased from BD Bioscience PharMingen (San Jose, CA, USA).

### Immunofluorescence Staining

Uterus were collected from mice after cardiac perfusion with PBS and fixed in 4% formaldehyde solution immediately and made into paraffin sections. Paraffin sections were stained with hematoxylin and eosin (HE). Paraffin sections of uterus were rehydrated and boiled in EDTA buffer (pH 6.0) for 20 min to induce antigen retrieval. After washing, tissue sections were blocked with 5% goat serum for 30 min at 37°C, followed by staining with rabbit anti-mouse CD3 antibody (Abcam, polyclone, 1:100) and armenian hamster anti-mouse γδT antibody (Santa Cruz Biotechnology, clone: UC7-13D5, 1:100) at 4°C overnight. Sections were washed four times for 5 min each time and incubated with Alexa Fluor 555-conjugated anti- rabbit IgG (Beyotime, 1:1000) plus DyLight 488-conjugated anti-Syrian hamster IgG (Abcam, 1:1000) for 30 min at 37°C in the dark. After a final washing, cover slips were mounted onto slides with fluoroshield mounting medium with DAPI (4, 6-diamidino-2-phenylindole, Abcam). Images were captured with Olympus microscope BX53 and processed with LSM Image Examiner software (Zeiss).

### RT-PCR

For the gene expression of chemokine CCL20 in uterus and blood, uterus and blood were collected as described above. Total RNA were extracted by Trizol reagent (Invitrogen) and then reverse-transcribed with a cDNA Synthesis Supermix (novoprotein scientific Inc) according to the manufacturer’s instruction. Amplification of cDNA was conducted in a DNA thermal cycler (Biometra, Germany). The following sense and antisense primers for each molecule were used: CCL20 Forward: 5′-CTCCTGGAGCTGAGAAT-3′, reverse: 5′-C ATCTTCTTGACTCTTAGGC-3′; glyceraldehyde-3-phosphate dehydrogenase (GAPDH) forward: 5′-TCAATGAAG GGGTCGTTGAT-3′, reverse: 5′-CGTCCCGTAGACAAAATGG T-3′. The ratio of CCL20 over GAPDH was calculated according to the relative intensities of the bands revealed under UV illumination with Bio-1D software (VilberLourmat, Marne la Vallee, France).

### Matrigel Invasion Assay

Transwell plates (6.5 mm in diameter) (Corning, Corning, NY, USA) containing polycarbonate filters with a pore size of 8.0 mm were used to perform the experiment. The upper surface of the filter was coated with 50 μg Matrigel (Corning, Corning, NY, USA) and dried aseptically. The Matrigel was rehydrated with 200 μl warm RPMI- 1640 with 1% BSA for 2 h before use. The isolated murine trophocytes (0.5 × 10^5^ in 200 μl RPMI-1640 with 1%BSA) were seeded in the upper chamber. rmIL-17 (R&D Systems, USA) at final concentrations of 0, 2, 20 and 100 ng/ml was added. The lower chamber was filled with 800 μl RPMI-1640 with 20% FBS. The cells were allowed to invade for 24 h in 5% CO2 at 37°C. The migrated cells on the lower surface were fixed in methanol for 10 min at room temperature, then stained with 0.1% crystal violet for 20 min and washed with PBS three times for 5 min each time. The cells that had migrated to the lower surface were counted under a light microscope in five fields at a magnification of ×200. The results are expressed as the ratio of the mean number of invasive cells with treatment relative to the controls.

### Statistical Analysis

Data were presented as the mean ± standard error of mean (SEM). Statistical significance was analyzed by Mann-Whitney test using Prism5 (GraphPad, San Diego, CA, USA). Significant p-values are indicated in figures for the following ranges: NS, no significance; *, P < 0.05; **, P <0.01; ***, P < 0.001; ****, P < 0.0001.

## Results

### γδT Cells Were Enriched in Uterus Compared to Blood and Most of Uterine γδT Cells Distributed in Endometrium

**Figure 1:**
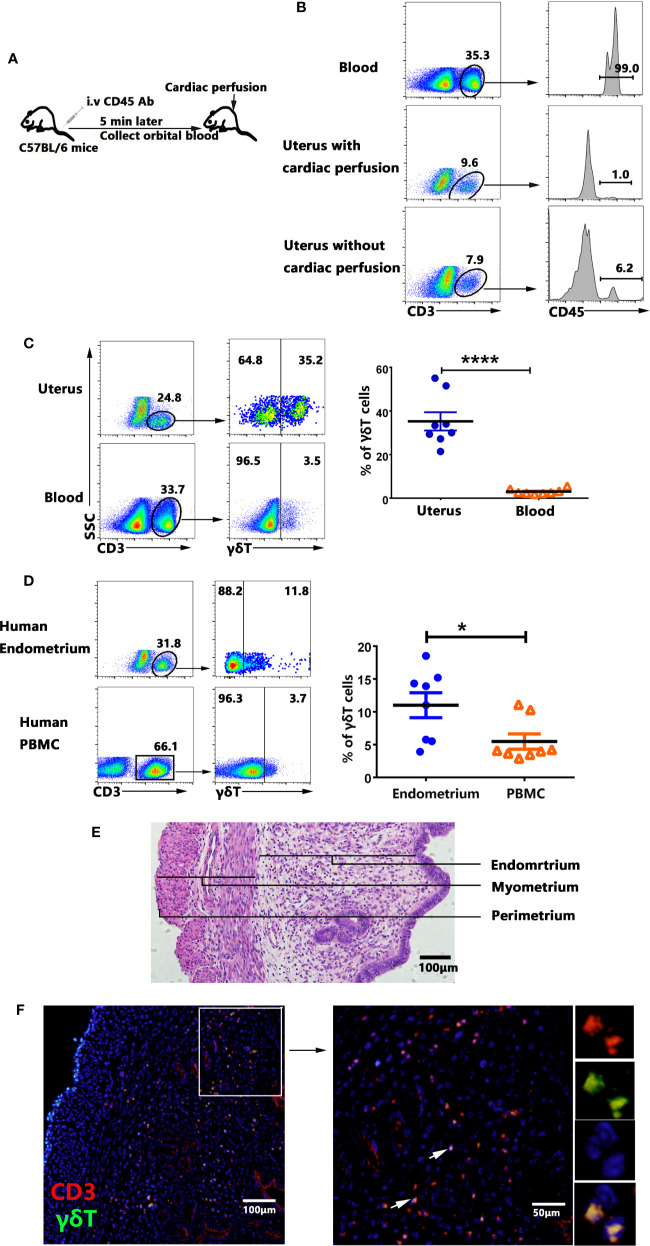
Percentages and tissue localization of γδT cells within uterus. Female mice were intravenously injected (i.v.) with CD45-PE antibody to label the circulating T cells, collected orbital blood after 5 min and removed the blood in tissues by cardiac perfusion **(A)**. The mononuclear cells from uterus and blood were stained with anti-CD3 and checked for the proportion of CD3^+^CD45^+^T cells by FACS. Data were representative of three independent experiments with 4 mice per group **(B)**. The representative graphs and statistical analyses for the percentages of γδT cells in uterus and blood were shown **(C)**. The representative graphs and statistical analyses for the percentages of γδT cells in human endometrium and peripheral blood were shown **(D)**. Plots were gated on total live γδT cells. Histology of uterus sections was stained with haematoxylin and eosin **(E)**. Immunofluorescence staining of CD3 (red), γδT cells (green) and DAPI (blue) in paraffin-embedded uterus tissues was shown **(F)**. The arrows display the colocalization of CD3 and γδT cells. The scale bars represented 100 μ M (left) and 50 μ M (right). The data of each point from six mice were expressed as the mean ± SEM. The statistical significance was determined with the Mann–Whitney U test. *P < 0.05, ****P < 0.0001.

To rule out the influence of peripheral blood γδT cells, female mice were intravenously injected (i.v.) with anti-CD45-PE antibody to label the circulating T cells, collected orbital blood 5 min later and removed the blood in tissues by cardiac perfusion (**Figure 1A**). The uterus was collected and the cells were prepared and checked for CD45^+^ cells compared with blood by flow cytometry. The results (**Figure 1B**) showed that 99% T cells in blood were labeled with CD45-antibody and only 1% of the T cells in uterus were labeled with CD45-antibody after cardiac perfusion. Approximate 6.2% T cells in uterus were CD45-antibody labeled without cardiac perfusion. The experiment proved that cardiac perfusion could effectively remove the blood in uterus and rule out the influence of blood T cells. We then compared the percentage of γδT cells in uterus and blood T lymphocytes. The result clearly showed that the percentages of γδT cells in uterus (34.0%) was significantly higher than those in blood (1.78%, p<0.0001) (**Figure 1C**). Similarly, the percentages of γδT cells in human endometrium was much higher than those in peripheral blood (**Figure 1D**). HE staining was used to show the structure of endometrium, myometrium and perimetrium (**Figure 1E**). To find out the distribution of γδT cells in uterus tissues, immunofluorescence analysis of paraffin uterus sections confirmed that most γδT cells including T cells in uterus distributed in endometrium, and a small part were distributed in the myometrium, but not in perimetrium (**Figure 1F**). These results indicated that γδT cells were enriched in uterus and most of uterine γδT cells distributed in endometrium.

### The Majority of γδT Cells in Uterus Are Tissue Resident Memory Cells

**Figure 2:**
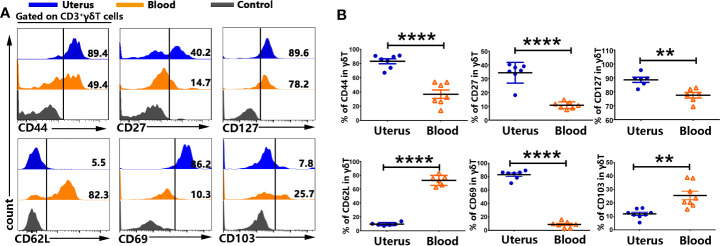
γδT cells from uterus expressed memory and residence markers. The mononuclear cells from uterus and blood were prepared and stained with anti-CD3, γδT, CD44, CD27, CD127, CD62L, CD69, and CD103, and assessed by FACS. Gated on CD3^+^γδT cells from uterus (blue) and blood (orange), compared to the control (gray), the representative graphs for the expression of CD44, CD27, CD127, CD62L, CD69, and CD103 were shown **(A)**. Statistical results **(B)** from 6-9 independent experiments with six mice in each experiment were shown as mean ± SEM, and the statistical significance was determined with the Mann–Whitney U test. **<0.01, ****P < 0.0001.

To compare the phenotypes between γδT cells from uterus and blood, we analyzed the expression of memory and residence markers by FACS after cell surface staining. The results showed that γδT cells from uterus expressed significantly higher levels of CD44, CD27 and CD127 compared to γδT cells from blood. Moreover, γδT cells from uterus expressed significantly lower levels of CD62L (9.6%) compared to those from blood (p<0.0001). In addition, we found that γδT cells from uterus expressed significantly higher levels of CD69 than γδT cells from blood (p<0.0001), but expressed lower levels of CD103 than γδT cells from blood (p<0.01) (**Figures 2A, B**). Taken together, our results demonstrated that the majority of γδT cells in uterus were tissue resident memory γδT cells.

### γδT Cells From Uterus Expressed Higher Levels of CCR6 and Lower Levels of CXCR3, CCR7 Compared to γδT Cells From Blood

**Figure 3:**
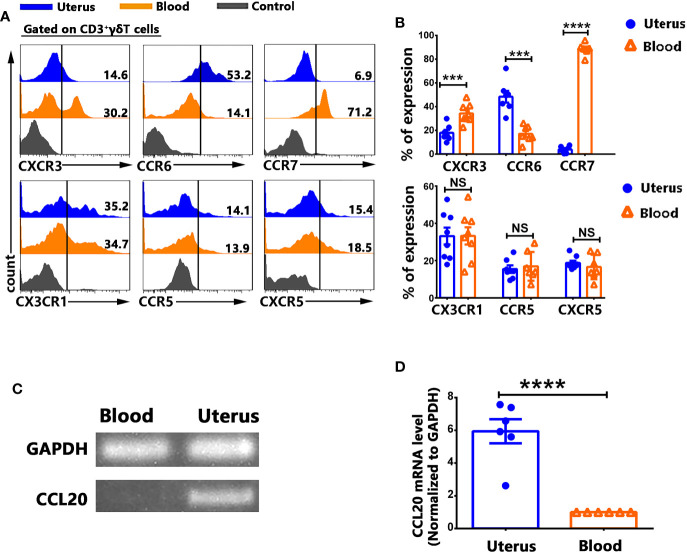
γδT cells from uterus expressed higher levels of CCR6 and lower levels of CXCR3, CCR7 than γδT cells from blood. Uterus and blood mononuclear cells were stained with anti-CD3, γδT, CXCR3, CCR6, CCR7, CX3CR1, CCR5, CXCR5, and analyzed by flow cytometry. The cells were gated on CD3^+^γδT^+^ and the representative histogram graph for the expression of CXCR3, CCR6, CCR7, CX3CR1, CCR5, and CXCR5 were shown **(A)**. Statistical results **(B)** were from 6-8 independent experiments with six mice in each experiment. The levels of CCL20 and GAPDH mRNA in uterus and in blood were determined by RT-PCR **(C)**, and the ratio of CCL20 to GAPDH were quantified by densitometry **(D)**. Data were shown as mean ± SEM. The statistical significance was determined with the Mann–Whitney U test. NS, no significance, ***P < 0.001, ****P < 0.0001.

In order to gain insight into the mechanisms of γδT cells recruitment to the uterus, the expression of chemokine receptors on γδT cells from uterus and blood were assessed by FACS. Results showed that γδT cells from uterus expressed higher levels of CCR6 but expressed lower levels of CXCR3 and CCR7 than γδT cells from blood (**Figures 3A, B**). There was no difference in the expression of CX3CR1, CCR5, CXCR5 on γδT cells from uterus and blood (**Figures 3A, B**). In addition, uterus expressed higher levels of CCL20 than in blood (p<0.0001) (**Figures 3C, D**). Overall, our results indicated that the CCR6-CCL20 chemokine axis might contribute to the selective migration of γδT cells into uterus.

### Tissue Resident γδT Cells in Uterus Expressed High Levels of IL-17 But Not IFN-γ

**Figure 4:**
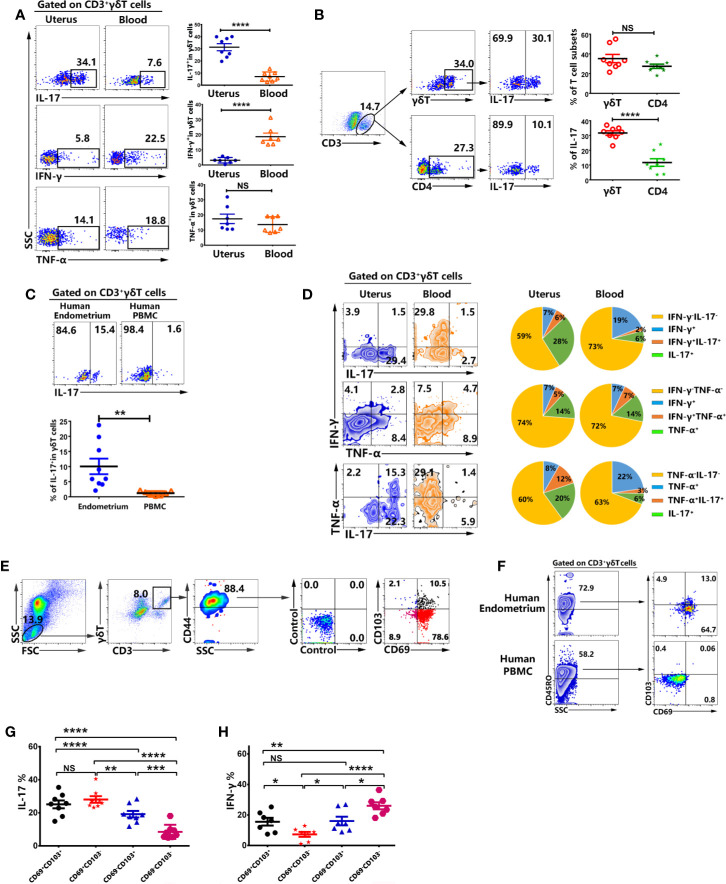
Tissue resident memory γδT cells from uterus expressed high percentages of IL-17 but less expression of IFN-γ. The mononuclear cells from uterus and blood were stimulated with or without PMA plus ionomycin in the presence of BFA for 6 h and analyzed by FACS. The representative graph and statistical results from 7-8 independent experiments with six mice in each experiment for the expression IL-17, IFN-γ and TNF-α in γδT cells were shown **(A)**. The representative graph and statistical results for the expression of IL-17 in γδT and CD4^+^T cells were shown **(B)**. The representative graph and statistical results for the expression of IL-17 in γδT cells from human endometrium and peripheral blood were shown **(C)**.The co-expression of IL-17, IFN-γ and TNF-α was analyzed by FACS. The representative graph and pie chat (n=6) of IL-17, IFN-γ and TNF-α single and co-expression were shown **(D)**. Gated on uterine CD3^+^γδT^+^CD44^+^ cells, the representative graph of the expression of CD69 and CD103 were shown **(E)**. Gated on CD3^+^γδT^+^ cells, the representative graph of the expression of CD45RO, CD69, and CD103 in human endometrium and peripheral blood were shown **(F)**. The expression of IL-17 **(G)** and IFN-γ **(H)** on CD69^+^CD103^+^, CD69^+^CD103^−^, CD69^−^CD103^+^, CD69^−^CD103^−^γδT cells from uterus was analyzed by FACS. The data were shown as mean ± SEM and statistical significance was determined with the Mann–Whitney U test. NS, no significance, *P < 0.05, **P < 0.01, ***P < 0.001 and ****P < 0.0001.

To evaluate the expression of IL-17, IFN-γ and TNF-α from γδT cells, the mononuclear cells from uterus and blood were stimulated with or without PMA plus ionomycin for 6 h in the presence of BFA. The results from FACS data demonstrated that higher percentages of γδT cells from uterus expressed IL-17 (31.7%) compared with γδT cells from blood (5.6%. p<0.0001), but lower levels of IFN-γ compared to blood (p<0.0001). Moreover, there was no difference on the expression of TNF-α (**Figure 4A**). In addition, our study further demonstrated that the proportion of γδT cells in uterus was equal to that of CD4^+^T cells. However, the expression of IL-17 by γδT cells (31.7%) was around three times more than that of CD4^+^T cells (11.7%) (**Figure 4B**). Consistent with results in mouse, γδT cells from human endometrium also expressed significantly higher levels of IL-17 compared to γδT cells from peripheral blood (**Figure 4C**). To examine the co-expression of IL-17, IFN-γ, and TNF-α between γδT cells from uterus and blood, we gated on γδT cells and the results showed that high percentages of uterine γδT cells expressed IL-17 and IL-17^+^γδT cells did not co-express IFN-γ but some IL-17^+^γδT cells co-expressed TNF-α. High percentages of blood γδT cells expressed IFN-γ and did not co-express with IL-17. As for the co-expression of IFN-γ and TNF-α, a large amount of TNF-α^+^γδT cells co-expressed IFN-γ both by uterus and blood (**Figure 4D**). Gated on uterine CD3^+^γδT^+^CD44^+^ cells, the results showed that the majority of γδT cells in uterus were CD69^+^CD103^+^, CD69^+^CD103^−^, or CD69^−^CD103^+^ (**Figure 4E**). Results in human also showed that most γδT cells in human endometrium were tissue resident memory T cells, highly expressing CD45RO, CD69, and CD103 (**Figure 4F**). To confirm the expression of cytokines by CD69^+^CD103^+^, CD69^+^CD103^−^, CD69^−^CD103^+^ and CD69^−^CD103^−^ cells, we gated on CD3^+^γδT^+^CD44^+^ cells and analyzed the expression of IL-17 and IFN-γ by FACS. Our results demonstrated that subsets of tissue resident γδT cells (CD69^+^CD103^+^, CD69^+^CD103^−^ and CD69^−^CD103^+^) expressed higher percentages of IL-17 and lower percentages of IFN-γ than CD69^−^CD103^−^γδT cells in uterus (**Figures 4G, H**).Taken together, these data indicated that tissue resident γδT cells expressed higher percentages of IL-17 than circulating γδT cells in uterus.

### γδT Cells From Uterus Expressed High Level of Cytotoxic Molecules Which Co-Expressed IFN-γ but not IL-17

**Figure 5:**
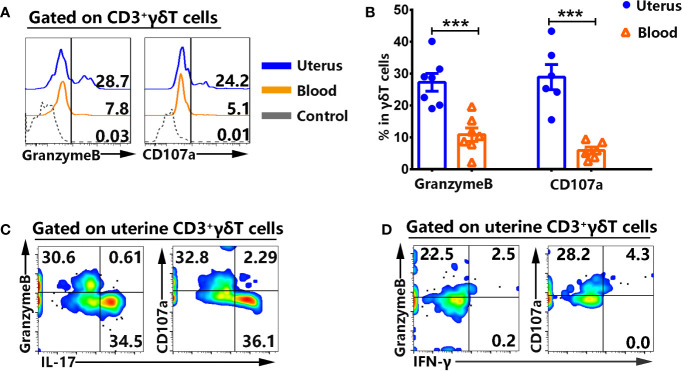
γδT cells from uterus highly expressed cytotoxic molecules and did not co-express IL-17. Uterus and blood mononuclear cells were stained, gated on CD3^+^γδT^+^ cells and analyzed by flow cytometry for the expression of cytotoxic molecules. The expression of GranzymeB and CD107a was assessed after intracellular and cell surface staining by FACS. The representative histogram graph **(A)** and statistical results **(B)** from 6-7 independent experiments with six mice in each experiment were shown. The mononuclear cells from uterus were stimulated with or without PMA plus ionomycin in the presence of BFA for 6 h and analyzed by FACS for the co-expression of IL-17, IFN-γ with GranzymeB and CD107a. The graphs were representative of six independent experiments **(C, D)**. The data were shown as mean ± SEM. The statistical significance was determined with the Mann–Whitney U test. ***P < 0.001.

The expression of cytotoxic molecules on γδT cells from uterus and blood were examined by FACS. Results showed that γδT cells from uterus expressed higher levels of GranzymeB (27.3%) than γδT cells from blood (10.9%, p<0.001), and higher percentages of CD107a (28.9%) than γδT cells from blood (5.9%, p<0.001) (**Figures 5A, B**). By contrast, there was no difference in the expression of NKG2D (data not shown). In addition, the results showed that GranzymeB^+^ and CD107a^+^γδT cells in uterus co-expressed IFN-γ but not IL-17 (**Figures 5C, D**).

### Transcription Factor RORγt but Not pSTAT3 Modulated the Expression of IL-17 in Uterus

**Figure 6:**
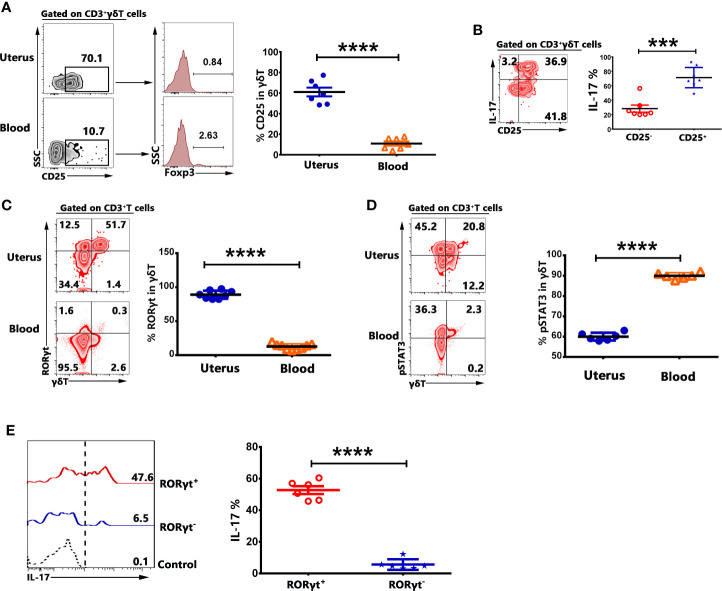
γδT cells in uterus expressed CD25 and transcription factors RORγt. The expression of CD25 and transcription factor Foxp3 on γδT cells from uterus and blood were assessed by FACS. Gated on γδT cells, the representative graph and statistical results for the expression of CD25 and Foxp3 were shown **(A)**. The mononuclear cells from uterus and blood were stimulated with or without PMA plus ionomycin in the presence of BFA for 6 h. The expression of IL-17 by CD25^+^ and CD25^−^γδT cells was analyzed and the representative graph as well as summary data were shown **(B)**. The expression of RORγt **(C)** and pSTAT3 **(D)** on γδT cells from uterus and blood was shown. The expression of IL-17 by RORγt^+^ and RORγt^-^γδT cells was analyzed and the representative graph and summary data were shown **(E)**. Data were expressed as the mean ± SEM, and each dot represented one independent experiment with six mice. The statistical significance was determined with the Mann–Whitney U test. ***P < 0.001 and ****P < 0.0001.

To investigate the possible mechanism of the expression of IL-17 by γδT cells in uterus, the mononuclear cells from uterus and blood were stimulated with or without PMA plus ionomycin for 6 h in the presence of BFA and stained with mAbs. The results demonstrated that 61.0% of γδT cells in uterus were CD25^+^ but CD25^+^γδT cells did not co-express Foxp3 (**Figure 6A**). In addition, IL-17 was mainly expressed by CD25^+^γδT cells in uterus (**Figure 6B**). The data showed that 90.0% of γδT cells in uterus and 12.7% of γδT cells in blood expressed transcription factor RORγt (**Figure 6C**). In contrary to RORγt, γδT cells in uterus expressed lower percentages of pSTAT3 than did γδT cells in blood (p<0.0001) (**Figure 6D**). Gated on uterine γδT cells, we found that IL-17 was mainly expressed by RORγt^+^γδT cells (**Figure 6E**). Those results indicated that γδT cells in uterus were highly activated and expressed transcription factor RORγt, which might be the reason why γδT cells in uterus could express IL-17.

### rmIL-17 Promoted the Invasion of Murine Trophocytes *In Vitro*

**Figure 7:**
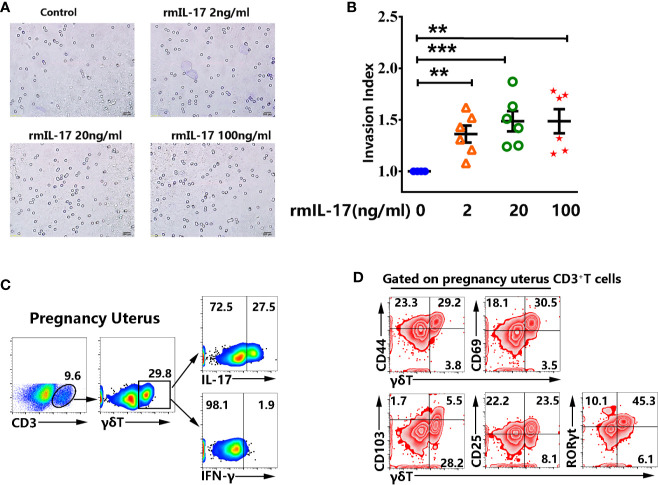
rmIL-17 promoted the invasion of murine trophocytes *in vitro*. The isolated trophocytes were cultured with different concentration of rmIL-17 (0, 2, 20, 100 ng/ml) at the upper chamber. The trophocytes migrated to the lower surface were photographed and the representative graphs were shown (×200) **(A)**. The statistical results **(B)** were expressed the ratio of the mean number of invasive cells with treatment related to the controls. Data were expressed as the mean ± SEM. The mononuclear cells from pregnancy uterus were stimulated with or without PMA plus ionomycin in the presence of BFA for 6 h. The representative graph for the proportion of γδT cells from pregnant uterus and production of IL-17 and IFN-γ were shown **(C)**. Gated on CD3^+^T cells in pregnant uterus, the representative graph for the expression of CD44, CD69, CD103, CD25, RORγt in γδT cells were shown **(D)**. Data were representative of three independent experiments with 6 mice per group. The statistical significance was determined with the Mann–Whitney U test. **P < 0.01, ***P < 0.001.

To determine the function of IL-17 on pregnancy, murine trophocytes were isolated at pregnancy day 13.5. We investigated the invasion of trophocytes after treatment with different concentration of rmIL-17. Importantly, the results showed that rmIL-17 could promote the invasion of trophocytes in a dose-dependent manner (**Figures 7A, B**). The enhancement of IL-17 in trophocytes invasion prompted us to confirm whether there was significant difference in non-pregnant and pregnant uterus. Similar to the results from non-pregnant mice, γδT cells were enriched in pregnancy uterus and highly expressed IL-17 but did not express IFN-γ (**Figure 7C**). γδT cells in pregnancy uterus also highly expressed memory marker CD44, residence marker CD69, activation marker CD25 and transcription factor RORγt, but expressed less CD103 (**Figure 7D**).

## Discussion

γδT cells are programmed into two main effector subsets that produce either interferon γ (IFN-γ) or interleukin 17 (IL-17) depend on the strength of the signal they encountered in fetal thymus (5, 6). These fetal-derived γδT cells are typically innate-like and seed peripheral tissues where they maintain for life. IL-17–producing γδT cells (γδ17 T cells) preferentially migrate to the genital tract, dermis and lungs. Fetal-derived γδ17 T cells are characterized as RORγt^+^CCR6^+^CD25^+^CD27^−^. γδ17 T cells are known to constitute a major source of IL-17 in various non-lymphoid tissues and interestingly contribute to disease development and normal tissue physiology in different tissues (8, 9, 24). γδ17 T cells are found to be widely distributed in maternal-fetal interface during pregnancy as well as in non-pregnant uterus (4). However, the role of γδ17 T cells in sustaining normal pregnancy remains unknown. In addition, there are emerging data point to a pathogenic role of IL-17 in pre-eclampsia, pre-term birth and miscarriage (25–27). It is urgent to comprehensively study the immunological characteristics and function of γδ17 T cells in pregnancy and non-pregnant uterus.

The γδT cells reached the 34% of T cells and it was equal to the proportion of CD4^+^T cells in uterus. By contrast, γδT cells in peripheral blood were much sparser than in uterus. Our study demonstrated that most of γδT cells and T cells in uterus were distributed in endometrium, and a small part were distributed in the myometrium, but not in perimetrium. The tissue localization of γδT cells within endometrium provided an ideal environment for both protecting against infections and regulating immune rejection response to allogenic fetus during pregnancy. γδT cells in uterus significantly expressed memory marker CD44 and expressed minimal CD62L and CCR7, indicating that they lack a capacity to adhere to HEV and hence they cannot circulate between peripheral tissues and lymphoid organs. Although γδT cells in uterus expressed higher levels of CD27 than γδT cells in blood, consistently with the study in adipose (8), γδ17 T cells were CD27^−^ and IL-17 did not co-express with CD27 (data not shown). CD69 and CD103 are markers commonly used to identify CD8^+^ or CD4^+^T_RM_ cells. However, studies in lungs after *Bordetella pertussis* and *Streptococcus pneumonia* infection showed that tissue resident memory γδT cells expressed CD69 and some co-expressed CD103, supporting the conclusion that CD69 and/or CD103 expression were also surface markers of tissue resident γδT cells (17, 28). Our result indicated that a considerable fraction of γδT cells in uterus expressed CD69 and some also co-expressed with CD103. Taken together, the majority of γδT cells in uterus were CD44^+^CD62L^−^CCR7^−^ CD69^+^γδT cells and displayed a tissue resident memory γδT cell phenotype.

It is well known that chemokines and their receptors act together with adhesion molecules to control the migration of lymphocytes to lymphoid and non-lymphoid tissues. Our results demonstrated that most of γδT cells in uterus significantly expressed CCR6 but less expressed CXCR3. Consistently with the high expression of CCR6, uterus expressed higher levels of CCL20 than in blood which is the only ligand to CCR6. It is widely recognized that CCR6 expression was clearly associated with the production of IL-17 in CD4^+^T cells and γδT cells (29, 30). Furthermore, IL-17–producing γδT cells were dependent on CCR6- recruitment into the injured liver and inflamed skin (31, 32). At least in part, CCR6-CCL20 chemokine axis is essential for uterine γδT cells to produce IL-17 and recruit into uterus. γδT cells in uterus expressed significantly higher levels of cytotoxic molecules GranzymeB and CD107a compared to γδT cells in blood. In addition, Granzyme B^+^ and CD107a^+^γδT cells did not express IL-17. It is possible that there are two γδT cell subsets with different functions in uterus, one is IL-17–producing γδT cells another one is cytotoxic γδT cells.

Over the past decades, IL-17 has received much attention for its pro-inflammatory role in autoimmune disease. However, a substantial body of research demonstrated that IL-17 had important context- and tissue-dependent roles in maintaining health during response to injury, physiological stress and infection (21, 33). Th17 cells is traditional considered to be the main source of IL-17. In recent years, studies have found that γδT cells constituted a major source of IL-17 in various non-lymphoid tissues (4, 8, 34). Our results indicated that after polyclonal activation, γδT cells in uterus expressed significantly higher levels of IL-17, lower levels of IFN-γ and equal levels of TNF-α compared to γδT cells in blood. Tissue resident γδT cells (CD69^+^CD103^+^, CD69^+^CD103^−^, and CD69^−^CD103^+^) in uterus expressed higher percentages of IL-17 than circulating γδT cells (CD69^−^CD103^−^). Transcription factors RORγt regulates the differentiation of γδ17 T cells and the production of IL-17. IL-6-STAT3 activation is crucial to turn on RORγt expression and development of Th17 T cells (35–37). Our results indicated that 70% of γδT cells in uterus expressed CD25 but did not express Foxp3, indicating CD25^+^γδT cells in uterus were not Foxp3^+^γδ Treg cells which were regulatory γδT cells and reported to be present in human PBMC and tumor infiltrating leukocytes(TIL) (38–41). In addition, nearly 90% of γδT cells in uterus expressed RORγt and 50% of RORγt^+^γδT cells expressed IL-17 but expressed lower percentages of pSTAT3 than γδT cells in blood. These data indicated that γδT cells were the major source of IL-17 and transcription factor RORγt but not pSTAT3 modulated the production of IL-17 in uterus.

It was reported that human trophocytes expressed high levels of IL-17 receptor and by secreting IL-17 Th17 cells could promote invasion of human first-trimester trophocytes (42). Clearly, our results confirmed that rmIL-17 could also promote the invasion of murine trophocytes *in vitro*. In addition, our study demonstrated that γδT were enriched in pregnancy uterus and there were no significant difference in cytokine production, memory and activation markers between non-pregnant and pregnant uterus. Taken together, our study for the first time showed that tissue resident memory γδT cells were enriched in uterus and expressed IL-17 but not IFN-γ, which could promote the invasion of trophocytes. In addition, γδT cells in uterus were highly activated and fully expressed transcription factor RORγt. These findings not only enrich knowledge on the immunological characteristics of γδT cells, but also lay the foundation for future research on uterine γδT cells in pregnancy and autoimmune disease.

## Data Availability Statement

The original contributions presented in the study are included in the article/supplementary materials. Further inquiries can be directed to the corresponding authors.

## Ethics Statement

The animal study was reviewed and approved by the Animal Ethics Committee, Sun Yat-sen University.

## Author Contributions

SK performed most experiments and analyzed data with the support from CW. QW performed flow cytometry on uterus. JH performed immunofluorescence staining. BY and CL contributed to scientific planning. PC and CW oversaw and designed the study. SK and CW wrote the manuscript. All authors contributed to the article and approved the submitted version.

## Funding

This study was supported by the National Natural Science Foundation of China (Grant No. 81971556).

## Conflict of Interest

The authors declare that the research was conducted in the absence of any commercial or financial relationships that could be construed as a potential conflict of interest.
